# *Staphylococcus microti* Strains Isolated from an Italian Mediterranean Buffalo Herd

**DOI:** 10.3390/ani13010182

**Published:** 2023-01-03

**Authors:** Monica Ambrosio, Francesca Paola Nocera, Francesca Garofalo, Pasquale De Luca, Alex Grinberg, Luisa De Martino

**Affiliations:** 1 Department of Veterinary Medicine and Animal Production, University of Naples ‘Federico II’, Via Delpino 1, 80137 Naples, Italy; 2 Istituto Zooprofilattico Sperimentale del Mezzogiorno, 80055 Portici, Italy; 3 Stazione Zoologica Anton Dohrn of Naples, Villa Comunale, 80121 Naples, Italy; 4 School of Veterinary Science, Massey University, Palmerston North 4442, New Zealand; 5 Task Force on Microbiome Studies, University of Naples ‘Federico II’, 80131 Naples, Italy

**Keywords:** *Staphylococcus microti*, buffalo milk, subclinical mastitis, intramammary infection, milking parlor surfaces

## Abstract

**Simple Summary:**

Non-aureus staphylococci (NAS) are frequently found in bovine milk samples as well as on teat skin associated with subclinical mastitis (SCM). *Staphylococcus microti* is a new species among NAS resulted to recently be the predominant species in the milk of water buffaloes (*Bubalus bubalis*) affected by intramammary infection (IMI). The aim of this study was to assess the occurrence of *S. microti* in milk and milking parlor equipment samples of an Italian buffalo farm. No clinical mastitis was noticed, but *S. microti*-associated SCM and IMI were detected. The presence of *S. microti* was also revealed on milking parlor surfaces. The results of the antimicrobial susceptibility tests highlighted a complete resistance to tetracyclines, and 20.4% of the isolated *S. microti* exhibited a multidrug-resistant (MDR) profile. In conclusion, the presence of this bacterium in the milk of buffaloes in the absence of evident mastitis clinical signs underlines the need for further studies, and its finding on milking parlor surfaces suggests that the environmental quality of the milking parlor plays an important role in influencing both *S. microti*’s spread and the microbial communities of the milk.

**Abstract:**

*S. microti* is a new species among non-aureus staphylococci (NAS) frequently found in bovine milk samples and associated with subclinical mastitis (SCM). The aim of this study was to analyze the presence of *S. microti* in 200 composite milk samples and 104 milking parlor surface swabs collected at a buffalo farm in Southern Italy to define its presence in milk and a milking parlor environment. The samples were inoculated onto different agar plates, and the isolates were identified by MALDI-TOF MS. The strains identified as *S. microti* (54/304 samples, 17.8%) were collected, and their purified genomic DNA was subjected to PCR amplification and whole 16S rRNA gene sequencing. Furthermore, their phenotypic resistance profiles were evaluated by a disk diffusion method, and the genotypic characterization of the tetracycline resistance was performed for the *tet*M and *tet*K genes by multiplex PCR. Four and forty-seven *S. microti* isolates from milk samples of lactating animals with subclinical mastitis (SCM) and intramammary infection (IMI), respectively, and three isolates from milking parlor surfaces were recovered. The genomic DNA was purified from the bacterial isolates, and the amplification and sequencing of the 16S gene further supported the proteomic identification as *S. microti*. No clinical mastitis was detected in the herd during the study period. The antimicrobial susceptibility testing revealed a worrisome 100% resistance to tetracyclines, genotypically mediated by the *tet*M gene for all strains. This study highlights that *S. microti* may be commonly isolated from dairy buffalo milk and milking parlor equipment. Its association with SCM or IMI remains to be established.

## 1. Introduction

The Mediterranean buffalo (*Bubalis bubalis*) is a large bovid widely distributed throughout Southern Italy. Buffalo milk has a high economic and social value in the Campania Region, as it is the raw material used to produce Mozzarella di Bufala Campana Protected Denomination of Origin (PDO), a worldwide renowned cheese.

Mastitis is one of the most economically important diseases affecting the dairy industry worldwide, and it is almost always caused by bacteria [1]. Thus, the identification of bacterial pathogens associated with clinical and subclinical mastitis is essential to understand the etiology of this disease, reduce animal culling and develop suitable therapy and preventative measures. According to the National Mastitis Council, the diagnosis of mastitis should be based on an interpretation of the somatic cell count (SCC) and bacteriological milk culture results [2].

The milk somatic cell count (SCC) is adopted as an indicator of udder health in single milk samples and is also important for monitoring farm hygiene in bulk milk. Clinically, cow’s milk samples with an SCC < 200 × 10^3^ cells/mL and negative to bacteriological culture are indicative of a healthy status. An SCC < 200 × 10^3^ cells/mL and a positive bacteriological culture is indicative of an intramammary infection (IMI), and an SCC > 200 × 10^3^ cells/mL and a positive bacteriological culture is indicative of a subclinical mastitis (SCM) or clinical mastitis, according to the absence or presence of clinical signs, respectively [3].

Staphylococci, especially the coagulase-positive *Staphylococcus aureus*, are among the pathogens associated with mastitis in farmed ruminants. The causative role of non-aureus staphylococci (NAS) has grown over the years, and today they are considered as etiological agents of mastitis in cows, goats, and sheep [4] and, in addition, they are more frequently isolated from milk [5]

More than 45 recognized NAS species have been described so far, and *S. microti* is a new species firstly described in 2010 [6]. Precisely, the first two strains of *S. microti* were isolated between 1999 and 2002 from the liver and kidneys of common voles (*Microtus arvalis* Pallas) in the Czech Republic [6]. Another strain was recovered from the skin of a small mammal (belonging to rodents or insectivores) in northeastern Poland [7]. Phylogenetic studies based on the sequence analysis of the 16S rRNA gene and several housekeeping genes revealed that *S. microti* is closely related to *S. rostri* and *S. muscae* [8]. Subsequently, eleven *S. microti* strains were identified in milk samples collected from dairy cows affected with clinical and subclinical mastitis characterized by a high self-curing rate [9]. The complete genomic sequence of the *S. microti* DSM 22,147 strain, isolated from the viscera of common voles, has been reported by Hu et al. [10], which described a genome without plasmids and with a GC content of 38% in a 2,381,859 bp long sequence.

Data on the presence and causal role of *S. microti* in cattle are scarce, but recently the species was found in a buffalo herd and was associated with cases of subclinical mastitis and alterations in milk protein composition [11].

In this study, we investigated the occurrence, supplemented by two identification analyses, of *S. microti* in buffalo milk and milking parlor surface samples of a buffalo farm in the Campania Region (Salerno, Italy). The strains’ in vitro susceptibility to commonly used antimicrobials was investigated, and a genotypic tetracycline resistance analysis was also performed. In addition, *S. microti*’s association with different clinical statuses of udders was evaluated.

## 2. Materials and Methods

### 2.1. Ethical Statement

This study was approved by the Institutional Animal Ethics Committee of the University of Naples Federico II (Italy) PG/2020/0092228 of 11/06/2020. All of the dairy buffaloes sampled in this study were from a single herd and were subjected to routine milk sampling for diagnostic purposes. Milk and milking parlor surface samples were collected with the owner’s permission, and no written informed consent was required.

### 2.2. Collection of Samples and Somatic Cell Count

The milk samples were collected from 3 to 5 year old lactating Mediterranean buffaloes in a herd of approximately 100 animals maintained in free-stall barns. The lactating buffaloes were milked twice a day in a tandem milking parlor. A total of 200 composite milk samples were collected aseptically over seven sampling periods from June to December 2020. For each monthly sampling, approximately 20 buffaloes in different stages of lactation were haphazardly chosen for the sampling. Only 23 animals were sampled two times with different sampling intervals in a range of 1–6 months. No clinical signs of mastitis were observed by the breeder for any of the sampled buffalo, and no antibiotic therapy was administered during the study period.

For the milk sampling, the first stream of milk was discharged, and two composite milk samples were collected from each lactating buffalo after the routine pre-milking cleaning during the evening milking and placed in sterile tubes (Thermo Fisher Scientific, Str. Rivoltana, Km 4-20090 Rodano, Milan, Italy). A total of 50 mL was collected for the somatic cell count (SCC) and 15 mL for the bacterial culture in two different tubes. The milk samples were kept at 4 °C and transported to the laboratory within 24 h. The SCC was performed using a NucleoCounter^®^ SCC-100™ (ChemoMetec Inc. 8950 Villa La Jolla Drive-Suite A127 La Jolla, CA, USA) at the Istituto Zooprofilattico Sperimentale del Mezzogiorno (Portici, Naples, Italy), with a measurement range of 1 × 10^4^ to 200 × 10^4^ SCC/mL, and the results are expressed as the number of cells per milliliter of milk. The milk composition was analyzed using a Milkoscan FT120 (Foss Electric A/S, Denmark), based on Fourier transform infrared (FTIR) spectra, to measure the fat, protein, and lactose content; a possible correlation between composition and SCC increase was determined.

Bacteriologically negative milk samples with <200 × 10^3^ cells/mL were considered healthy (H). Differently, bacteriologically positive milk samples were categorized in three groups considered diseased: (1) samples with intramammary infection (IMI) presenting SCCs < 200 × 10^3^ cells/mL; (2) samples with subclinical mastitis (SCM) presenting SCC values > 200 × 10^3^ cells/mL in the absence of clinical signs; (3) samples with clinical mastitis (CM) presenting SCC values > 200 × 10^3^ cells/mL in the presence of clinical signs.

In addition, 104 swabs were collected from the surfaces of the milking parlor, such as the milking boxes and milking unit. Each swab was placed in Stuart W/O CH transport medium (Aptaca Spa, Asti, Italy) and transferred within 24 h to the laboratory for bacteriological examination.

### 2.3. Bacterial Isolation and Identification

All bacteriological examinations were performed at the Microbiology Laboratory of the Department of Veterinary Medicine and Animal Production of the University of Naples “Federico II” following an internal protocol. Precisely, the microbiological count method was the standard plate count agar method, and 50 μL of each milk sample was diluted in 1 mL of buffered peptone water, and 200 μL of inoculum was spread on each of the following plates: Mac Conkey Agar, 5% Columbia Sheep Blood agar, Mannitol Salt Agar, and Saboraud Dextrose Agar (Oxoid, Milan, Italy). The same plates were used for the surface swabs. All plates were incubated for 24 h at 37 °C in aerobic conditions, and then the phenotypically equal colonies from the milk samples were counted to perform an enumeration of the CFUs.

The isolation of 1 to 3 different colony types and ≥500 CFU/mL for each colony type was considered as a positive sample, whereas milk samples for which more than 3 colony types and/or <500 CFU/mL colonies of any bacterial colony was considered as a contaminated sample. To obtain pure cultures, single colonies were subcultured on Columbia Sheep Blood agar and incubated as above; then, they were identified by matrix-assisted laser desorption/ionization-time of flight mass spectrometry (MALDI-TOF MS) (Bruker Daltonics Inc., Bremen, Germany). Score values below 1.7 indicated a nonreliable identification, between 1.7 and 1.99 a probable genus identification and equal or above 2.0 a certain genus identification and probable or highly probable species identification. A bacterial test standard (BTS) (Bruker Daltonics, Germany) was used as a calibrator for quality control.

### 2.4. Sequencing of 16S rRNA Gene

The 16S ribosomal gene was amplified from each DNA and then entirely sequenced. The genomic DNA was extracted from the overnight solid cultures of each identified *S. microti* strain using the commercial Isolate II Genomic DNA kit (Bioline, London, UK) according to the manufacturer’s instructions. The quantity and quality of the DNA were assessed by spectrophotometric reading of the A260/A280 ratio (Eppendorf BioPhotometer 6131). The DNA samples were stored at −20 °C until use. Table 1 shows all of the primers used in this study. We designed the primers Smi16-11F, Smi16-750F, Smi16Fdown, Smi16-337R, Smi16Rup, Smi16-1072R and Smi16-1527R based on the preliminary sequencing data and on the reference sequence MF678892.1 found in GenBank. The other primers were retrieved from the cited literature. We first tested the presence of *S. microti* in our samples by amplifying the genomic DNA with the primers Smi16F and Smi16R (20 ng each DNA, 2 mM each dNTP and 0.3 υM each primer, 5U Takara PrimeSTAR GXL DNA polymerase, Takara Bio, Inc., San Jose, CA, USA). Amplification protocol: initial denaturation at 98 °C 3 min, 35 cycles at 98 °C 10 s, 60 °C 15 s, 68 °C 1 min, obtaining a fragment of 370 bp, which sequence matched the *S. microti* and *S. rostri* 16S rRNA genes. We attempted to identify the whole 16S gene using 2 generic bacterial primers, B27F and B1492R (20 ng each DNA, 2 mM each dNTP and 0.7 µM each primer, 5U Takara PrimeSTAR GXL DNA polymerase, Takara Bio, Inc., San Jose, CA, USA). Amplification protocol: initial denaturation at 98 °C 3 min, 35 cycles at 98 °C 10 s, 57 °C 15 s, 68 °C 1.5 min and a final step at 68 °C 5 min. The amplified product of approximately 1400 bp was then sequenced from its terminal regions using internal primers: Smi16F, Smi16Fdown, Smi16Rup and Smi16R. The sequence obtained showed that we amplified a single fragment that matched exactly the *S. microti* 16S rRNA gene. Based on our sequencing data and on the reference sequence in GenBank, MF678892.1, we designed the primers Smi16-11F and Smi16-1527R, which encompassed almost the whole rDNA 16S sequence, except for the first 10 bp. With these two primers, we were able to amplify the whole 16S gene from each DNA sample (20 ng each DNA, 2 mM each dNTP and 0.3 uM each primer, 5U Takara PrimeSTAR GXL DNA polymerase, Takara Bio, Inc., San Jose, CA, USA). Amplification protocol: initial denaturation at 98 °C 3 min, 35 cycles at 98 °C 10 s, 60 °C 15 s, 68 °C 1.5 min and a final step at 68 °C 5 min, obtaining a 1516 bp product in each case. Each PCR product was sequenced with all of the primers listed in Table 1 using the Brilliant Dye Terminator kit v3.1 (Nimagen, The Netherlands) and run on the 3700 xl DNA analyzer (Thermo Scientific, Waltham, MA, USA). The obtained sequences were assembled with SeqMan II sequence analysis software (DNASTAR Inc., Madison, WI, USA) independently for each of the 1511 bp amplicons.

### 2.5. Antimicrobial Susceptibility Testing of S. microti Isolates

All of the *S. microti* isolates were assessed for the following panel of antibiotics: amoxicillin–clavulanate (AMC, disk content: 20/10 µg), amikacin (AK, disk content: 30 µg), cefoxitin (FOX, disk content: 30 µg), clindamycin (CD, disk content: 2 µg), ciprofloxacin (CIP, disk content: 5 µg), erythromycin (E, disk content: 15 µg), gentamicin (CN, disk content: 10 µg), kanamycin (K, disk content: 30 µg), oxacillin (OX, disk content: 1 µg), oxytetracycline (T, disk content: 30 µg), penicillin (P, disk content: 10 IU), streptomycin (S, disk content: 10 µg), sulfamethoxazole–trimethoprim (SXT, disk content: 25 μg) and tetracycline (TE, disk content: 30 µg). As reported in Table 2, the chosen antibiotics belonged to eight classes. Based on the Kirby–Bauer method, inoculated Muller–Hinton agar plates were incubated at 37 °C for 24 h in an aerobic atmosphere. The isolates were classified as susceptible (S), intermediate (I) or resistant (R) according to the Clinical and Laboratory Standards Institute [15] and to the European Committee on Antimicrobial Susceptibility Testing [16] guidelines. *S. microti* isolates nonsusceptible to at least one antibiotic in more than two families were considered multidrug-resistant strains, according to Magiorakos et al. [17].

### 2.6. Genotypic Characterization of Tetracycline Resistance

Resistance to tetracyclines was further investigated in *S. microti*-isolated strains by performing multiplex PCR for the *tet*M and *tet*K genes. It has been reported that tetracycline resistance is mediated above all by these two genes in NAS isolated from bovine subclinical or clinical mastitis cases [18,19]. The *Tet*M gene encodes for ribosomal protection proteins, which reduce the affinity of tetracycline for the ribosome, and the *tet*K gene encodes for efflux proteins, which prevent tetracycline from accumulating inside the cell. Multiplex PCR was performed for all 54 *S. microti* isolates, as already described by Ullah et al. [20]. The list of primers and the multiplex PCR conditions used for amplification of tetracycline resistance genes are described in Table 3. For all of the multiplex PCR reactions, there was always a positive (DNA from a clinical *S. aureus* strain positive to *tet*M and *tet*K genes) and a negative (without bacterial DNA) control. Multiplex PCR was performed using Biorad T100TM Thermo cycler (BioRad, Hercules, CA, USA).

### 2.7. Data Analysis

The interest ratios among the variable categories were compared statistically using the two-tailed Fisher’s exact test (GraphPad Software Inc., Avenida De La Playa La Jolla, CA, USA).

## 3. Results

### 3.1. Somatic Cell Count (SCC) Results

The results of the bacteriological analysis in relation to the SCCs of the milk samples are shown in Table 4. Among all of the samples, 54 (27%) were defined as ‘healthy’, having an SCC < 200 × 10^3^ cells/mL and no bacterial growth, while 124 (62%) and 22 (11%) exhibited bacterial growth and variable SCC values. Precisely, among the milk samples obtained from animals without clinical signs, 62% had an SCC < 200 × 10^3^ cells/mL and bacterial growth, classified as IMI, whereas 11% of the samples presented SCC values > 200 × 10^3^ cells/mL and bacterial growth compatible with the definition of SCM. No animals with clinical signs of mastitis were observed during the study period.

Furthermore, the two-tailed Fisher’s exact test evidenced a value statistically significant with *p* < 0.05 between the two dependent variables: bacterial growth (presence or absence) and values of SCC (≤200 × 10^3^ cells/mL or >200 × 10^3^ cells/mL) in milk samples, as reported in Table 4.

### 3.2. S. microti and Somatic Cell Count (SCC) Correlation

White and weakly hemolytic, catalase-positive, coagulase-negative and oxidase-negative colonies were identified as *S. microti* by MALDI-TOF analysis, with a log(score) ≥ 2.0 in all identifications, indicating a reliable identification at the species level.

The *S. microti* isolation was revealed in 51/200 (25.5%) milk samples. Only 23 animals were sampled two times with different sampling intervals, and precisely 10 showed positivity to *S. microti* in both samplings, with a maximum interval of 6 months, and 13 animals resulted positive to *S. microti* in one of the two samplings. Furthermore, 18 animals positive to *S. microti* were sampled only once.

The evaluation of the SCC for each milk sample revealed the presence of *S. microti* in four milk samples (4/22, 18.2%) with an SCC value > 200 × 10^3^ cells/mL, and 47 strains were isolated from the milk samples (47/124, 37.9%) with an SCC value < 200 × 10^3^ cells/mL, compatible with SCM and IMI, respectively. *S. microti* were isolated alone or in combination with other bacteria, as described for the *S. microti*-associated SCM cases (Table 5). As demonstrated in Table 5, the MALDI-TOF identification of *S. microti* always presented an optimal score > 2.0, only 11/38 (28.9%) identifications of other bacterial species showed a slightly <2.0 score. The supplementary data (Table S1) show that all 47 *S. microti*-associated IMI cases exhibited a high level of genus and species identification. Moreover, 3/104 (2.9%) *S. microti* were isolated from surface swabs in two different samplings, as well as precisely two strains from a milking unit and one from a milking box, which presented a good score of identification (>2.0), as reported in Table S2.

### 3.3. Sequencing of 16S rRNA Gene

The 16S rRNA genes were amplified and sequenced from genomic DNA purified from the bacterial isolates with the primers listed in Table 1. The sequences were analyzed with the BLAST algorithm (National Center for Biotechnology Information, NCBI, Bethesda, MD, USA) against the Nucleotide Collection database, retrieving the *S. microti* 16S gene (accession MF678892.1, 99.28% identity).

### 3.4. Antimicrobial Susceptibility Testing and Genotyping Characterization of Tetracycline Resistance in S. microti Strains

**Figure 1:**
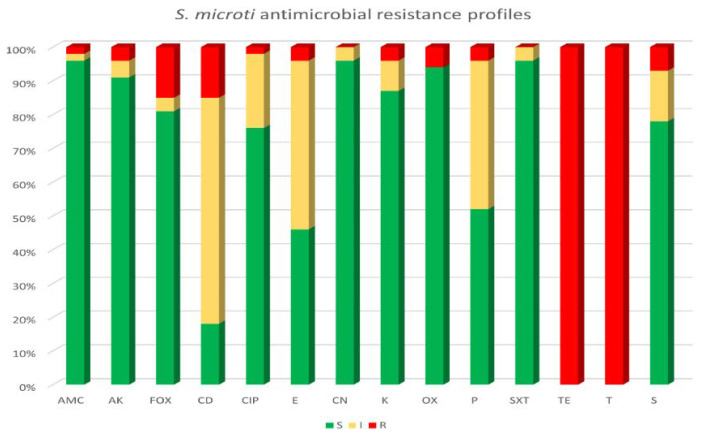
Antibiotic resistance profiles of 54 *S. microti* isolates. Tested antibiotics: amoxicillin–clavulanate (AMC), amikacin (AK), cefoxitin (FOX), clindamycin (CD), ciprofloxacin (CIP), erythromycin (E), gentamicin (CN), kanamycin (K), oxacillin (OX), penicillin (P), sulfamethoxazole–trimethoprim (SXT), tetracycline (TE), oxytetracycline (T) and streptomycin (S).

The antimicrobial susceptibility testing results highlighted a complete resistance to both tetracycline and oxytetracycline (100%) for all 54 *S. microti* isolated strains, while the majority of the strains (52/54, 96.5%) were susceptible to amoxicillin–clavulanate, gentamicin and sulfamethoxazole–trimethoprim, followed by amikacin (90.7%, 49/54), kanamycin (87%, 47/54), streptomycin (77.8%, 42/54) and ciprofloxacin (76%, 41/54). Referring to *beta*-lactams antibiotics, high levels of susceptibility were observed for oxacillin and cefoxitin, being 94.4% (51/54) and 81.5% (44/54), respectively; but nearly half of the isolated strains were resistant to penicillin (51.9%, 28/54). Clindamycin and erythromycin resulted to be the antibiotics with the highest number of strains showing intermediate susceptibility (66.7%, 36/54 and 50%, 27/54, respectively). Figure 1 shows the antimicrobial susceptibility pattern of all bacterial isolates obtained in this study, classifying them as sensitive (S), intermediate (I) or resistance (R).

In addition, an important finding concerns the prevalence of *S. microti* strains with a multidrug-resistance (MDR) profile, as described in Table 6. In fact, 20.4% (11/54) of the total isolates showed resistance to at least three classes of antibiotics, and all were isolated from the milk samples.

In addition, the genotypic characterization of tetracycline resistance revealed the presence of the *tet*M gene in all collected *S. microti* strains. No *tet*K gene was found.

### 3.5. Analysis of the Major Constituents of Milk

Data on the nutritional composition of almost all of the milk samples evidenced optimal values as fat (ranging between 7.0 and 8.0 g/100 g), protein (ranging between 3.0 and 4.0 g/100 g) and lactose (4.0 and 5.0 g/100 g) (data not shown). Only one milk sample, exhibiting a positive bacteriological examination with the growth of *S. microti* and *Citrobacter freundii* and an SCC > 200 × 10^3^ cells/mL, showed a slight decrease in the value of the tested milk constituents (i.e., fat, protein and lactose).

## 4. Discussion

Bacteria of the genus *Staphylococcus* have been considered the main etiological agents associated with cases of SCM [21,22] and CM [23] in buffaloes. However, overall, non-aureus staphylococci (NAS) species are currently the most prevalent pathogens causing SCM, IMI and, rarely, CM in dairy buffaloes [24,25]. The predominance of NAS in milk samples is remarkable [26,27], and their ability to cause inflammatory processes should not be underestimated. Together with microbial analysis, the somatic cell counts (SCCs) contained in milk can provide an accurate evaluation of the milk quality. Based on this principle, in this study, we considered milk samples with an SCC < 200 × 10^3^ cells/mL and negative to bacteriological culture as a healthy status of the buffaloes, and milk samples with an SCC < 200 × 10^3^ cells/mL and a positive bacteriological culture indicative of IMI, and an SCC > 200 × 10^3^ cells/mL with a positive bacteriological culture as SCM cases, as already reported by Puggioni et al. [3]. Moreover, animals with clinical signs, SCC values > 200 × 10^3^ and positive for bacteriological examination were defined as CM cases [3].

The SCC level changed over time through the course of the cases, and the lack of this information represents a limitation of this study; in fact, only 23 animals were sampled twice, and relevant changes were not observed in the SCC values between the two sampling periods.

Our study performed in a buffalo farm determined a statistically significant higher level of IMI (62%) than SCM (11%) cases, with no cases of CM. Several studies have analyzed the prevalence of SCM in buffalo populations around the world, reporting a prevalence of approximately 26.2% and 37.6% in India and Bangladesh, respectively [28,29]. The meta-analysis work conducted by Krishnamoorthy et al. [30] on numerous studies published worldwide during the period 1967–2019 has identified values of 46% and 32% for SCM cases in buffaloes worldwide and in India, respectively. In addition, it has been reported that in SCM mastitis, the percentage of culture-negative milk samples ranged from 28.6% [31] to 38.6% [32], whereas in our study, all milk samples with a high SCC value (>200 × 10^3^ cells/mL) were bacteriologically positive. Our rate of 11% SCM agrees with values between 5 and 20% that were reported by Joshi and Gokhale [33]. In contrast, the percentage of IMI cases that we detected was 62%, a percentage higher than the 40.4% reported by Singha et al. [34] but closer to the value of 63% reported by Moroni et al. [5].

Values of 7.1% or 19.7% have been reported for healthy milk samples with an SCC < 200 × 10^3^ cells/mL and a negative bacteriological examination [3,35], whereas in our study healthy samples were 27%.

The bacterial identification performed in this study, among the isolated NAS, highlighted a new species known as *S. microti*, a bacterial species that is still not well known on buffalo farms. In fact, the identification of *S. microti* has been only once reported as the predominant species in the milk of water buffaloes with SCM [11]. Previously, a first case of IMI in cattle was reported in Poland [9] and more recently in a bovine herd in Germany, where *S. microti* was detected as the causative pathogen of IMI in combination with other NAS [36]. To the best of our knowledge, there are no published studies investigating the presence of *S. microti* at the same time in milk and milking parlors of buffalo farming. Thus, for the first time, we isolated 54 strains of *S. microti* over seven samplings, with an average of eight strains per sampling, and no prevalence of isolation between the summer and autumn periods was observed. Moreover, we obtained in 10/41 animals the isolation of *S. microti* in two different samplings. Precisely, 51 strains were isolated from milk samples and 3 from the milking parlor surfaces, highlighting the predominant presence of this strain in the milk samples.

It is known that sequencing and taxonomic resolution based on 16S rRNA gene alone are limited [37], and a cost-effective alternative are taxonomic analyses using MALDI-TOF. Ribosomal RNA and ribosomal proteins can be used for phylogenetic analysis, being universally conserved in both eukaryotic and prokaryotic cells [38]. The identification of *S. microti*, here obtained by MALDI-TOF analysis, was perfectly confirmed by the classification method based on the complete sequence analysis of the 16S rRNA gene. However, MALDI-TOF analysis is a time-saving method for the routine identification of bacteria and can achieve 96% accuracy [39].

In addition, *S. microti* isolates were evaluated for an antibiotic susceptibility profile and all bacteria isolated both from the SCM and IMI cases were found to be 100% resistant to tetracycline and oxytetracycline. The susceptibility to other antibiotics agreed with a previous study that reported a high susceptibility rate to a panel of antibiotics, not including tetracyclines, tested for *S. microti* strains isolated from bovine milk samples [9]. In our study, 20.4% (11/54) of the *S. microti* isolates, showing resistance to three or more classes of antibiotics, were defined as MDR. Further molecular investigations are needed for four strains, which resulted to be resistant to cefoxitin but susceptible to penicillins, while two strains were oxacillin-resistant but susceptible to penicillin and cefoxitin. The MDR profile was not detected in the *S. microti* isolates from the milking parlor surfaces, which exhibited resistance to the tetracycline class and one of them also resistance to lincosamides. Currently, to our knowledge, there are no other studies on the genotypic characterization of tetracycline resistance in *S. microti* strains from buffalo. In our study, all 54 isolates showed the presence of the *tet*M gene, justifiable by their isolation performed at the same farm. Previously, the detection of both the *tet*K and *tet*M genes in NAS strains has been reported [40,41], as well as in *S. aureus* isolated from cows with mastitis [42].

The hygienic status of animals is very important to ensure healthy and quality milk, and both microorganisms and SCC should be minimized in raw milk [43]. The monitoring of IMI, SCM, CM and cleaning milking parlor routines represent an important aspect to profitable livestock economics worldwide.

From this study, we can conclude that the pathological and epidemiological roles of *S. microti* need further investigation as well as its association with IMI or SCM. Furthermore, new information on the presence of this bacterium in Italian buffalo-breeding herds, especially in the Campania Region where buffalo herds represent 80% of the national buffalo assets and is an important zootechnical and economic reality, is very important. In the context of the surveillance of NAS strains and of studies on antibiotic resistance profiles, including resistance gene identification, future work should also be focused on the detection and monitoring of MDR *S. microti* in wider areas of the Campania Region.

## 5. Conclusions

The present study revealed the occurrence and antibiotic resistance of *S. microti* strains isolated from buffalo milk samples and milking parlor surfaces. A limitation of this study is represented by the research performed only in one buffalo herd; thus, further surveillance studies are required to better understand the spread of this bacterial species in buffalo farms and particularly in lactating buffalo and milking parlors. Furthermore, the role of this bacterium and its pathological and epidemiological aspects need further investigation, as well as its association with IMI or SCM.

## Figures and Tables

**Table 1:** Primers used for the PCR sequencing of the 16S ribosomal gene.

Primer	Sequence	Reference
Smi16-11F	GGCGGCGTGCCTAATACATG	This study
Smi16-750F	GTGGGGATCAAACAGGAT	This study
Smi16F	CCTCTTCGGAGGACAAAGTGA	[12]
Smi16Fdown	GAATACGTTCCCGGGTCTTG	This study
Smi16-337R	CTGCTGCCTCCCGTAGG	This study
Smi16Rup	ATCCTGTTTGATCCCCAC	This study
Smi16-1027R	TCACTTTGTCCTCCGAAGAGG	This study
Smi16R	GACCCGGGAACGTATTCACC	[12]
Smi16-1527R	TAGAAAGGAGGTGATCCAGC	This study
B27F	AGAGTTTGATCMTGGCTCAG	[13]
B1492R	TACCTTGTTACGACTT	[14]

**Table 2:** Antibiotic classes tested to define the antimicrobial susceptibility profiles of the isolates.

Antibiotics	Disk Content	Antibiotic Class	Reference for Breakpoints
Amoxicillin–clavulanate (AMC)	20/10 µg	Penicillins	[15]
Penicillin (P)	10 IU		
Oxacillin (OX)	1 µg		
Amikacin (AK)	30 µg	Aminoglycosides	[15]
Kanamycin (K)	30 µg		
Gentamicin (CN)	10 µg		
Streptomycin (S)	10 µg		
Cefoxitin (FOX)	30 µg	Cephalosporins	[16]
Ciprofloxacin (CIP)	5 µg	Quinolones	[15]
Clindamycin (DA)	2 µg	Lincosamides	[15]
Erythromycin (E)	15 µg	Macrolides	[15]
Tetracycline (TE)	30 µg	Tetracyclines	[15]
Oxytetracycline (T)	30 µg		
Sulfamethoxazole–trimethoprim (SXT)	25 µg	Sulfonamides	[16]

**Table 3:** Primer sequences, amplicon size and amplicon program of *tet*M and *tet*K genes.

Gene	Primer Sequences (5’-3’ Sense and Antisense)	Amplicon Size (bp)	Amplifications Program
*tet*M	F: AGTTTTAGCTCATGTTGATG R: TCCGACTATTTAGACGACGG	1862	94 °C 15 s; 94 °C 1 min, 52 °C 1 min, 72 °C 90 s, for 30 cycles; 72 °C 5 min
*tet*K	F: GTAGCGACAATAGGTAATAGT R: GTAGTGACAATAAACCTCCTA	360	

**Table 4:** Bacterial culture results and somatic cell counts (SCCs) in buffalo milk samples.

Bacterial Culture	SCC Values (cell/mL)	Status *	No. of Samples/ 200 Samples	%	Fisher’s Two-Tailed
No bacterial growth	SCC ≤ 200.000	H	54	27.0%	*p* < 0.05
	SCC > 200.000	SCM	0	0%	
Bacterial growth	SCC ≤ 200.000	IMI	124	62%	*p* < 0.05
	SCC > 200.000	SCM	22	11%	

* H (healthy), SCM (subclinical mastitis) and IMI (intramammary infection).

**Table 5:** Bacterial species and MALDI-TOF scores in 22 buffalo milk samples showing high somatic cells counts (SCC) compatible with the definition of subclinical mastitis (SCM).

Sample	SCC/mL	Identified Bacterial Strains (Colony Forming Unit, CFU)	MALDI-TOF Score
1	2 × 10^5^	*Aeromonas hydrophila* (3000 CFU/mL)	*2.11*
2	2.9 × 10^5^	*Acinetobacter johnsonii* (5000 CFU/mL) *Staphylococcus simulans* (1000 CFU/mL)	*2.26* *2.20*
3	2.5 × 10^5^	*Staphylococcus microti* (10,000 CFU/mL) *Citrobacter freundii* (10,000 CFU/mL)	*2.07* *1.96*
4	2.8 × 10^5^	*Escherichia coli* (5000 CFU/mL) *Staphylococcus simulans* (1000 CFU/mL) *Staphylococcus sciuri* (500 CFU/mL)	*2.09* *2.20* *2.09*
5	2 × 10^6^	*Streptococcus agalactiae* (6000 CFU/mL) *Lactococcus lactis* (1100 CFU/mL) *Aeromonas hydrophila* (5000 CFU/mL)	*2.10* *2.17* *1.88*
6	2.7 × 10^5^	*Aeromonas hydrophila* (3000 CFU/mL)	*2.12*
7	5.3 × 10^5^	*Streptococcus agalactiae* (10,000 CFU/mL) *Corynebacterium xerosis* (1000 CFU/mL)	*2.27* *2.04*
8	3.6 × 10^5^	*Staphylococcus microti* (3000 CFU/mL) *Aerococcus viridans* (2000 CFU/mL) *Rothia endophytica* (200 CFU/mL)	*2.04* *1.79* *1.91*
9	2.9 × 10^5^	*Aerococcus viridans* (2000 CFU/mL)	*1.81*
10	2.7 × 10^5^	*Staphylococcus microti* (4000 CFU/mL)	*2.04*
11	2.3 × 10^5^	*Escherichia coli* (4000 CFU/mL)	*2.16*
12	2.9 × 10^5^	*Pseudomonas aeruginosa* (4000 CFU/mL)	*2.34*
13	2.2 × 10^5^	*Streptococcus agalactiae* (3000 CFU/mL)	*2.43*
14	2.5 × 10^5^	*Rothia amarae* (6000 CFU/mL)	*2.05*
15	5.1 × 10^5^	*Escherichia coli* (2800 CFU/mL) *Rothia amarae* (500 CFU/mL) *Aerococcus viridans* (700 CFU/mL)	*2.22* *1.94* *1.82*
16	2.9 × 10^5^	*Escherichia coli* (400 CFU/mL) *Streptococcus agalactiae* (5400 CFU/mL) *Aerococcus viridans* (200 CFU/mL)	*2.14* *2.29* *1.99*
17	1.5 × 10^6^	*Escherichia coli* (2000 CFU/mL) *Aerococcus viridans* (1100 CFU/mL)	*2.17* *2.14*
18	8.3 × 10^5^	*Streptococcus agalactiae* (5000 CFU/mL)	*2.19*
19	2.1 × 10^5^	*Staphylococcus microti* (200 CFU/mL) *Aerococcus viridans* (100 CFU/mL) *Escherichia coli* (500 CFU/mL)	*2.06* *1.89* *2.31*
20	4.5 × 10^5^	*Aerococcus viridans* (400 CFU/mL)	*1.90*
21	8.8 × 10^5^	*Streptococcus agalactiae* (4700 CFU/mL)	*2.04*
22	2.3 × 10^5^	*Aerococcus viridans* (4800 CFU/mL)	*1.99*

**Table 6:** Antibiotic resistance profiles of 11 multidrug-resistant *S. microti* strains.

	Penicillins			Aminoglycosides				Cephalosporins	Quinolones	Lincosamides	Macrolides	Tetracyclines		Sulfonamides
*S. microti* Strains	AMC	P	OX	AK	K	CN	S	FOX	CIP	DA	E	TE	T	SXT
7				R							R	R	R	
11							R	R		R		R	R	
12								R		R		R	R	
13	R	R						R				R	R	
14								R		R		R	R	
15								R		R		R	R	
16				R						R		R	R	
17									R	R		R	R	
19			R		R							R	R	
20		R						R				R	R	
22			R				R				R	R	R	

## Data Availability

Not applicable.

## Supplementary Materials

The following are available online at https://www.mdpi.com/article/10.3390/ani13010182/s1, Table S1: Bacterial species identification by MALDI-TOF of 47 milk samples positive to *S. microti* growth with or without other bacterial species. All milk samples presented somatic cell count (SCC) < 200 × 10^3^ cells/mL indicative of intramammary infection. Table S2: *S. microti* and other bacterial species isolated from 3 milking surface samples.
