# Supplementary material for: Staphylococcus microti Strains Isolated from an Italian Mediterranean Buffalo Herd
Source: Animals (Basel). 2023 Jan 3;13(1):182. doi: 10.3390/ani13010182 (PMC9817920; doi:10.3390/ani13010182)
Supplement: Supplementary file 1 [file animals-13-00182-s001.zip › Table S1.pdf]

Table S1 – Bacterial species identification by MALDI-TOF of 47 milk samples positive to S. *microti* growth with or without other bacterial species. All milk samples presented somatic cell count (SCC) <200 x 10^3 cells/mL indicative of intramammary infection.

Sample number	SCC/ml	Identified bacterial strains (colony forming unit, CFU)	MALDI-TOF score
1	1.7 x 10^5	*Staphylococcus microti* (3000 CFU/mL)	2.03
2	3.5 x 10^4	*Staphylococcus microti* (6000 CFU/mL)	2.02
3	4.2 x 10^4	*Staphylococcus microti* (4000 CFU/mL)	2.07
4	1.6 x 10^4	*Staphylococcus microti* (600 CFU/mL)	2.14
5	6.2 x 10^4	*Staphylococcus microti* (2000 CFU/mL)<br>*Escherichia coli* (500 CFU/mL)	2.08<br>2.23
6	8.7 x 10^4	*Staphylococcus microti* (800 CFU/mL)<br>*Klebsiella oxytoca* (500 CFU/mL)<br>*Acinetobacter proteolyticus* (500 CFU/mL)	2.01<br>2.15<br>2.16
7	2.9 x 10^4	*Staphylococcus microti* (2000 CFU/mL)<br>*Citrobacter freundii* (1500 CFU/mL)	2.05<br>2.22
8	7.3 x 10^4	*Staphylococcus microti* (600 CFU/mL)<br>*Raoultella ornithinolyca* (1000 CFU/mL)	2.06<br>2.27
9	9.2 x 10^4	*Staphylococcus microti* (3100 CFU/mL)<br>*Escherichia coli* (500 CFU/mL)<br>*Staphylococcus saprophyticus* (1400 CFU/mL)	2.02<br>2.47<br>1.96
10	3.7 x 10^4	*Staphylococcus microti* (500 CFU/mL)<br>*Aeromonas hydrophila* (2000 CFU/mL)	2.09<br>2.28
11	4.8 x 10^4	*Staphylococcus microti* (1100 CFU/mL)<br>*Staphylococcus xylosus* (500 CFU/mL)<br>*Aeromonas media* (700 CFU/mL)	2.07<br>2.23<br>1.70
12	1.1 x 10^4	*Staphylococcus microti* (2000 CFU/mL)<br>*Escherichia coli* (500 CFU/mL)<br>*Macrococcus caseolyticus* (500 CFU/mL)	2.08<br>2.27<br>2.06
13	1 x 10	*Staphylococcus microti* (700 CFU/mL)	2.06
14	8 x x 10^4	*Staphylococcus microti* (1500 CFU/mL)<br>*Aeromonas hydrophila* (1000 CFU/mL)	2.04<br>2.12
15	2.5 x 10^4	*Staphylococcus microti* (1500 CFU/mL)	2.04
16	2.8 x 10^4	*Staphylococcus microti* (1500 CFU/mL)<br>*Aeromonas hydrophila* (500 CFU/mL)	2.01<br>2.11
17	1.1 x 10^4	*Staphylococcus microti* (500 CFU/mL)	2.01
18	2.8 x 10^4	*Staphylococcus microti* (1200 CFU/mL)<br>*Escherichia coli* (1000 CFU/mL)<br>*Enterococcus aquimarinus* (800 CFU/mL)	2.12<br>2.26<br>1.76
	5.7 x 10^4	*Staphylococcus microti* (600 CFU/mL)	2.02
19	2.9 x 10^4	*Staphylococcus microti* (800 CFU/mL)<br>*Aerococcus viridans* (200 CFU/mL)<br>*Enterococcus aquimarinus* (1500 CFU/mL)	2.08<br>1.80<br>1.84
20	1.5 x 10^5	*Staphylococcus microti* (2000 CFU/mL)	2.10
21	5.8 x 10^4	*Staphylococcus microti* (2000 CFU/mL)	2.01
22	6.4 x 10^4	*Staphylococcus microti* (1000 CFU/mL)	2.01

23	1.5 x 10^5	*Staphylococcus microti* (900 CFU/mL)<br>*Aerococcus viridans* (600 CFU/mL)<br>*Lactococcus lactis* (100 CFU/mL)	2.10<br>1.96<br>2.00
24	3.1 x 10^4	*Staphylococcus microti* (1000 CFU/mL)<br>*Escherichia coli* (200 CFU/mL)<br>*Streptococcus uberis* (700 CFU/mL)	2.12<br>2.29<br>2.11
25	1 x 10^4	*Staphylococcus microti* (600 CFU/mL)	2.04
26	1.1 x 10^5	*Staphylococcus microti* (500 CFU/mL)<br>*Escherichia coli* (2500 CFU/mL)<br>*Aerococcus viridans* (100 CFU/mL)	2.09<br>2.13<br>1.80
27	4.8 x 10^4	*Staphylococcus microti* (500 CFU/mL)<br>*Staphylococcus epidermidis* (500 CFU/mL)	2.07<br>2.19
28	5.1 x 10^4	*Staphylococcus microti* (600 CFU/mL)	2.01
29	1.4 x 10^5	*Staphylococcus microti* (800 CFU/mL)<br>*Microbacterium oxydans* (400 CFU/mL)	2.08<br>2.03
30	2.6 x 10^4	*Staphylococcus microti* (1000 CFU/mL)<br>*Staphylococcus sciuri* (1200 CFU/mL)<br>*Aerococcus viridans* (800 CFU/mL)	2.01<br>2.12<br>1.99
31	7.2 x 10^4	*Staphylococcus microti* (16000 CFU/mL)<br>*Bacillus licheniformis* (1100 CFU/mL)<br>*Macrococcus caseolyticus* (1100 CFU/mL)	2.06<br>1.99<br>2.01
32	1.1 x 10^5	*Staphylococcus microti* (800 CFU/mL)<br>*Escherichia coli* (1400 CFU/mL)<br>*Acinetobacter towneri* (300 CFU/mL)	2.06<br>2.24<br>1.99
33	5.1 x 10^4	*Staphylococcus microti* (1100 CFU/mL)<br>*Staphylococcus chromogenes* (600 CFU/mL)<br>*Lactococcus lactis* (1300 CFU/mL)	2.01<br>2.11<br>1.87
34	1.5 x 10^4	*Staphylococcus microti* (1000 CFU/mL)<br>*Staphylococcus chromogenes* (800 CFU/mL)<br>*Aerococcus viridans* (1000 CFU/mL)	2.08<br>2.10<br>1.99
35	8.1 x 10^4	*Staphylococcus microti* (500 CFU/mL)<br>*Bacillus licheniformis* (800 CFU/mL)<br>*Aerococcus viridans* (2900 CFU/mL)	2.02<br>2.01<br>1.98
36	2.2 x 10^4	*Staphylococcus microti* (1600 CFU/mL)<br>*Aerococcus viridans* (1800 CFU/mL)	2.08<br>2.11
37	3.9 x 10^4	*Staphylococcus microti* (2000 CFU/mL)<br>*Bacillus licheniformis* (1200 CFU/mL)<br>*Aerococcus viridans* (1200 CFU/mL)	2.08<br>1.99<br>2.12
38	9.6 x 10^4	*Staphylococcus microti* (2500 CFU/mL)<br>*Bacillus licheniformis* (200 CFU/mL)	2.12<br>2.01
39	2.8 x 10^4	*Staphylococcus microti* (1200 CFU/mL)<br>*Escherichia coli* (500 CFU/mL)<br>*Bacillus licheniformis* (200 CFU/mL)	2.12<br>2.12<br>2.01
40	1.5 x 10^4	*Staphylococcus microti (1200 CFU/mL)*<br>*Streptococcus uberis (600 CFU/mL)*<br>*Acinetobacter indicus (200 CFU/mL)*	*2.08*<br>*2.23*<br>*1.77*
41	6.6 x 10^4	*Staphylococcus microti (2400 CFU/mL)*<br>*Acinetobacter indicus (500 CFU/mL)*<br>*Bacillus licheniformis (1700 CFU/mL)*	*2.02*<br>*1.77*<br>*1.88*
42	1.1 x 10^5	*Staphylococcus microti (700 CFU/mL)*<br>*Aerococcus viridans (4300 CFU/mL)*<br>*Escherichia coli (800 CFU/mL)*	*2.09*<br>*2.03*<br>*2.24*

43	1.6×10^5	*Staphylococcus microti (800 CFU/mL)*<br>*Escherichia coli (7000 CFU/mL)*	*1.90*<br>*2.19*
44	8.4×10^4	*Staphylococcus microti (1000 CFU/mL)*<br>*Escherichia coli (2000 CFU/mL)*	*2.20*<br>*2.25*
45	1.6×10^5	*Staphylococcus microti (600 CFU/mL)*<br>*Aerococcus viridans (1100 CFU/mL)*	*1.95*<br>*1.98*
46	3.3×10^4	*Staphylococcus microti (200 CFU/mL)*<br>*Escherichia coli (300 CFU/mL)*<br>*Aerococcus viridans (900 CFU/mL)*	*2.09*<br>*2.24*<br>*1.88*
47	1.2×10^5	*Staphylococcus microti (30000 CFU/mL)*<br>*Aerococcus viridans (600 CFU/mL)*	*2.05*<br>*2.09*
