# Supplementary material for: Staphylococcus microti Strains Isolated from an Italian Mediterranean Buffalo Herd
Source: Animals (Basel). 2023 Jan 3;13(1):182. doi: 10.3390/ani13010182 (PMC9817920; doi:10.3390/ani13010182)
Supplement: Supplementary file 1 [file animals-13-00182-s001.zip › Table S2.pdf]

Table S2- – *S. microti* and other bacterial species isolated from 3 milking surface samples

Sample number	Identified bacterial strains (number of colonies forming units, CFU)	MALDI-TOF score
1	***Staphylococcus microti*** (3400 CFU/mL)<br>*Bacillus licheniformis* (3000 CFU/mL)	2.07<br>1.88
2	***Staphylococcus microti*** (4800 CFU/mL)<br>*Escherichia coli* (1600 CFU/mL)<br>*Acinetobacter indicus* (4200 CFU/mL)	2.14<br>2.23<br>1.83
3	***Staphylococcus microti*** (2000 CFU/mL)<br>*Acinetobacter towneri* (600 CFU/mL)<br>*Corynetobacter xerosis* (1000 CFU/mL)	2.15<br>1.99<br>1.78
